# Xiaoyao Pills Prevent Lipopolysaccharide-Induced Depression by Inhibiting Inflammation and Protecting Nerves

**DOI:** 10.3389/fphar.2019.01324

**Published:** 2019-11-13

**Authors:** Boyu Shi, Jie Luo, Yang Fang, Xiaobo Liu, Zhili Rao, Rong Liu, Nan Zeng

**Affiliations:** Department of Pharmacology, College of Pharmacy, Chengdu University of TCM, Chengdu, China

**Keywords:** Xiaoyao pills, lipopolysaccharide, depression, inflammation, hippocampus, cortex

## Abstract

Lipopolysaccharides are pro-inflammation mediators that can induce inflammation in the serum, hippocampus, and cortex of animals. And lipopolysaccharide-induced neuroinflammatory state resulted in significant depression-like behaviors, including reduced locomotor activity in the open field test, reduced saccharin preference, added immobility time in tail suspension test and forced swimming test, decreased comb time in the splash test, and increased latency to food in the novelty suppressed feeding test time, and reduced the levels of neurotrophic factors and synaptic proteins, and decreased Nissl bodies. Treatment with Xiaoyao Pills ameliorated the depression-like behavior, decreased the levels of inflammatory indicators, increased those of neurotrophic factors and synaptic proteins, and restored Nissl bodies. Our study suggests that lipopolysaccharides induce inflammation and nerve injury, thereby leading to depression. Xiaoyao Pills could be considered a potential therapeutic candidate for inflammation-induced depression.

## Introduction

Depression is one of the most common mental illnesses. It has many pathogenic factors, which are related to gene and environment. Clinically, patients with depression show cognitive impairment and declining social capacity. According to the World Health Organization, by 2020, depression is predicted to become one of the main health burdens and the second most common disease reducing the affected individuals’ ability to work (Kessler et al., 2003). At present, the clinical treatment of depression mainly focuses on neurochemical and neurobiological mechanisms (Kupfer et al., 2012). Physiological inflammatory changes are closely correlated to depression as such, inflammation is now regarded as a sign of depression. Secretion of more proinflammatory cytokines [such as interleukin 6 (IL-6) and tumor necrosis factor alpha (TNF-α)] are reported in both patients with depression and animal models of depression (Gold, 2015). These cytokines affect neurotransmission and plasticity in the brain and suppress neurogenesis. Bacterial endotoxin lipopolysaccharide (LPS) challenge stimulates the acute inflammatory response and leads to depressive symptoms in humans and rodents. LPS challenge is commonly used to study inflammation-induced depression-like behaviors (Shi et al., 2017). However, conventional treatment usually relieves symptoms in less than 50% of patients (Casacalenda et al., 2002; El-Hage et al., 2013). Therefore, it is necessary to explore the pathogenesis of depression and find new potential antidepressants. At the same time, the treatment cost of depression should also be considered in order to reduce the economic burden on patients.

Many studies have shown that traditional Chinese medicines have potential antidepressant effects that complement or may even replace existing treatment methods (Kessler et al., 2002). XiaoYao San is a prescription for Xiaoyao Pills and is included in the Chinese Pharmacopoeia (National Pharmacopoeia Committee, 2015). They are prepared from XiaoYaoSan Decoction, and are more convenient to take, with equal efficacy. XiaoYaoSan decoction is a classical Chinese medicine formula that comprises eight herbal medicines (100 g of *Bupleurum chinense* DC., 100 g of *Angelica sinensis* Diels., 100 g of *Paeonia lactiflora* Pall., 100 g of *Atractylodes macrocephala* Koidz., 20 g of *Mentha haplocalyx* Briq., 100 g of *Poria cocos* Wolf., 80 g of *Glycyrrhiza uralensis* Fisch., 100 g of *Zingiber officinale* Rosc.). According to the theory of traditional Chinese medicine, XiaoYaoSan has been most frequently used to treat anxiety and depression by smoothing the liver, strengthening the spleen, and nourishing the blood (Zhang et al., 2012). Although there are few reports on the antidepressant effect of XiaoYao pill, the antidepressant effects of XiaoYaoSan and its improved formula Danzhi-XiaoYao-San have been confirmed. XiaoYaoSan improves the anxiety-like behaviors which might be related to the JNK signaling pathway in the hippocampus (Zhao et al., 2017). XiaoYaoSan can exert antidepressant effects by regulating the glutamate/glutamine cycle and the glutamate transporter GLT-1 and the counterpoise kynurenine metabolism pathway (Ding et al., 2017; Wang et al., 2017). The modified Danzhi-XiaoYao-San formula has been shown to improve depression-like behavior by reducing inflammatory factor levels (Zhu et al., 2015), inhibiting hyperactivity of the hypothalamic pituitary adrenal axis and regulating monoamine and amino acid neurotransmitters (Wu et al., 2016). The modified Xiaoyaosan (MXYS) can improve hippocampal neurogenesis and play an antidepressant role by regulating cerebral oxygen-dependent functional magnetic resonance imaging (fMRI) signals in mice (Gao et al., 2018). Many studies suggest that XiaoYao San has a role in improving depression-like behavior, but its antidepressant mechanism still remains unclear. Based on our previous studies, we speculate that XiaoYao San can exert antidepressant effects by inhibiting neuroinflammation and neuroprotection.

## Materials and Methods

### Drugs and Reagents

Xiaoyao Pills (XYW) (TaiJi, China), fluoxetine hydrochloride (FLX) (Patheon, France) and Amitriptyline hydrochloride (Sigma, USA) were dissolved in 0.9% saline to prepare solutions. Paeoniflorin (C_23_H_28_O_11_), Liquiritin (C_21_H_22_O_9_), Glycyrrhizic acid (C_42_H_62_O_16_), Ligustilide (C_12_H_14_O_2_) (Cheng Du Ai Fa, China). LPS (*Escherichia coli* 055:B5) was provided by Sigma (St. Louis, MO). Rabbit anti-TrkB antibody (1:1000; Cell Signaling Technology; Cat. No. #4603), rabbit anti-cAMP response element-binding protein (CREB) antibody (1:1000; Cell Signaling Technology; Cat. No. #9197S), rabbit anti-p-CREB antibody (1:1000; Cell Signaling Technology; Cat. No. #9198S), rabbit anti-β-Tubulin antibody (1:1000; Cell Signaling Technology; Cat. No. #2128). Rabbit anti-brain-derived neurotrophic factor (BDNF) antibody (1:1000; Abcam; Cat. No. #ab108319). Rabbit anti-DLG4, PSD95-specific, polyclonal antibody (1:1000; Proteintech; Cat. No. #20665-1-AP) and rabbit anti-synaptophysin polyclonal antibody (1:1000; Proteintech; Cat. No. #17785-1-AP). Rabbit anti-GAPDH antibody (1:1000; Servicebio; Cat. No. #GB11002). Rabbit anti-IL-6 polyclonal antibody (1:300; Servicebio; Cat. No. #GB11117) and Alexa Fluor 488 goat anti-rabbit IgG (1:400; Servicebio; Cat. No. #GB25303).

### Animals

Eight-week-old male C57BL/6J mice and Male Sprague-Dawley rats that weighed 220–240 g were purchased from Chengdu Dashuo. All animals were raised in standard cages in a room of constant temperature and humidity (22 ± 1′C; 40–60%) with a 12:12 dark/light cycle (lights on at 8:00 a.m.; off at 8:00 p.m.). The animals were given free access to food and water throughout the experiment except during the sucrose preference test (SPT) and novelty suppressed feeding test (NSFT). The experimental procedures were under the guidelines of the Committee for Animal Care and Use of Laboratory Animals, College of Pharmacy, Chengdu University of Traditional Chinese Medicine.

### Surgical Procedures

After acclimation, the rats were anesthetized and the guide cannulas (RWD Life Science Co., Ltd., Shenzhen, China) were implanted into lateral ventricle [anteroposterior (AP): -1.00 mm from the bregma; lateral (LM): 1.5 mm from the sagittal suture; deep ventricula (DV): 4.5 mm in depth relative to the skull]. The animal’s head was shaved off and fixed to the stereotaxis, and the incisor bar set at 4.5 mm below the interaural line. At the end of the operation, a stylet of the same length as the guide cannula is inserted to prevent obstruction. The rats were allowed to recover in their cages for 10 days.

### Xiaoyao Pills Quality Control

Xiaoyao Pill is composed of eight Chinese herbal medicine (Bupleurum chinense DC., Angelica sinensis Diels., Paeonia lactiflora Pall., Atractylodes macrocephala Koidz., Mentha haplocalyx Briq., Poria cocos Wolf., Glycyrrhiza uralensis Fisch., Zingiber officinale Rosc.), that each herbal medicine contains various ingredients in different contents. At present, there are few studies on the composition analysis of Xiaoyao Pill. The Chinese Pharmacopoeia only provides technical regulations for paeoniflorin (C_23_H_28_O_11_), which paeoniflorin content should not be less than 4.0 mg in 1.0 g of concentrated pills. But according to reports in the literature (Su and Yin, 2011; Yi et al., 2011; Cheng and Song, 2012; Bai et al., 2013), they analyzed the composition of Xiaoyao Pills, including Paeoniflorin, Liquiritin, Glycyrrhizic acid, Ligustilide, etc. In this study, we used HPLC to analyze the components of Paeoniflorin (C_23_H_28_O_11_), Liquiritin (C_21_H_22_O_9_), Glycyrrhizic acid (C_42_H_62_O_16_) and Ligustilide (C_12_H_14_O_2_).

**Figure 1:**
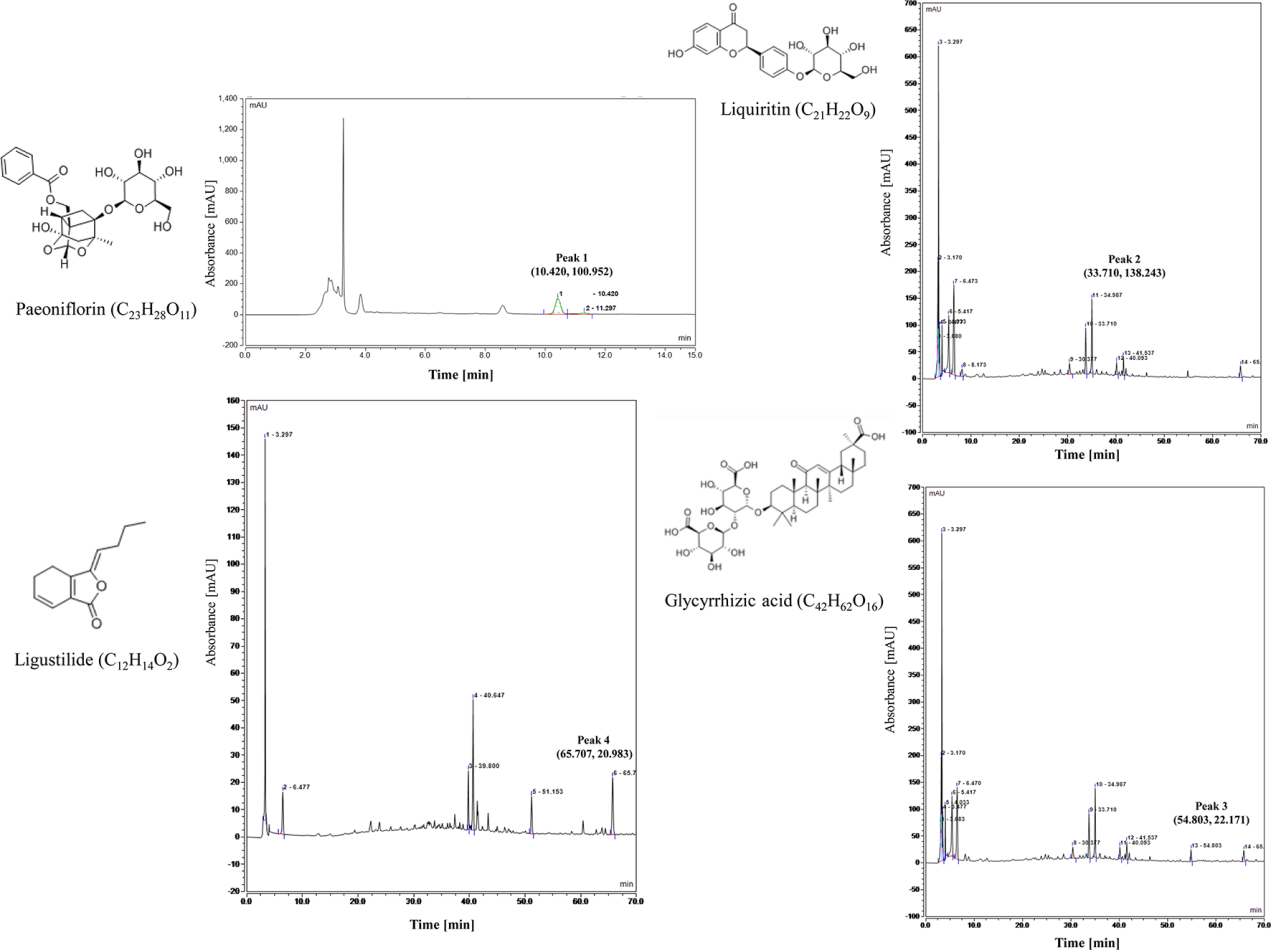
Control characteristic map (chromatogram) of Xiaoyao Pills (peak 1, peak 2, peak 3, peak 4 represent peak characteristic of Paeoniflorin, Liquiritin, Glycyrrhizic acid, Ligustilide, respectively.).

The analysis was performed by High Performance Liquid Chromatography (HPLC) (Thermo, USA). the column was C18 column (4.6×250 mm, 5 µm) and the chromatographic separation conditions were as follows: Column temperature: 30′C; Flow rate: 1.0 ml/min; Mobile phase: acetonitrile + 0.1% phosphoric acid (15:85); Detection wavelength: 230 nm; Stock solutions of Xiaoyao Pills was prepared by dissolving 0.4 g of analyte in 25 ml dilute ethanol. Content in Xiaoyao Pills was determined by quantitation Paeoniflorin (C_23_H_28_O_11_), Paeoniflorin content should not be less than 4.0 mg in 1.0 g of concentrated pills (National Pharmacopoeia Committee, 2015). The control characteristic map (chromatogram) of Xiaoyao Pills is shown in **Figure 1**, peak1 represent peak characteristic of Paeoniflorin. The content of Paeoniflorin in 1.0 g Xiaoyao Pill is 27.5 mg.

We further analyzed the active components in Xiaoyao Pills by High Performance Liquid Chromatography (HPLC) (Thermo, US). The column was C18 column (4.6 × 250 mm, 5 µm) and the chromatographic separation conditions were as follows: Column temperature: 35′C; Flow rate: 1.0 ml/min; Mobile phase: acetonitrile (A) + 0.1% phosphoric acid (B) (0∼10min, 5%A; 10∼25min, 5%A → 15%A; 25∼70 min, 15%A → 60%A); Detection wavelength: 280 nm (27-45 min, Liquiritin), 276 nm (45-60 min, Glycyrrhizic acid), 350 nm (60-70 min, Ligustilide); Stock solutions of Xiaoyao Pills was prepared by dissolving 1.0 g of analyte in 100 ml dilute methanol. Content in Xiaoyao Pills was determined by quantitation Liquiritin (C_21_H_22_O_9_), Glycyrrhizic acid (C_42_H_62_O_16_), Ligustilide (C_12_H_14_O_2_). The control characteristic map (chromatogram) of Xiaoyao Pills is shown in **Figure 1**; peak 2, 3, 4 represent peak characteristic of Liquiritin, Glycyrrhizic acid, Ligustilide, respectively. The content of Liquiritin, Glycyrrhizic acid, Ligustilide in 1.0 g Xiaoyao Pill is 1.0, 1.6, 0.4 mg, respectively.

### Drugs and Administration

Animals in the XYW group were intragastrically administered XYW (483.6 mg·kg^-1^, 0.93 g·kg^-1^, 1.86 g·kg^-1^) daily for 14 days prior to LPS injection. Animals in the FLX group (3.03 mg·kg^-1^, 10 mg·kg^-1^) daily for 14 days prior to LPS injection. Animals in the AMT group (Amitriptyline, 10 mg·kg^-1^) daily for 14 days prior to LPS injection. Meanwhile, animals in the control and LPS groups were intragastrically administered equivoluminal saline daily to ensure isocaloric intake.

All injections were performed on free-moving animals using a 5 µl microsyringe. The pro-inflammatory cytokine-inducer LPS was diluted to 100 ng·µl^-1^ with saline and was infused intracerebroventricularly at a dose of 100 ng/rat (flow rate: 0.5 µl·min^-1^) (Tang et al., 2016). Animals received a total of 300 ng LPS or saline throughout the experimental period.

### Behavioral Measurements

Mice were divided into two groups: one group was made to undergo the tail suspension test (TST) and the other the forced swimming test (FST). After 24 h of treatment, mice were tested for tail suspension. The mice were suspended with tape about 2.5 cm from the tail tap and 40 cm above the ground. The experiment lasted for 6 min and the mice were recorded for analysis. In the last 4 mins of the test, two observers recorded the immobility time. Before the FST, mice were placed individually into glass cylinders (height 30 cm, diameter 16 cm) containing 25 cm water maintained at 23–25′C. The formal test lasted for 6 min, and a mouse was judged to be immobile when it floated in an upright position and made only small movements to keep its head above water. The duration of immobility was recorded during the last 4 min of the testing period (Shi et al., 2017).

After 1 week of self-recovery, the rats from each treatment group were put into the corner of the open box. After 2 min of adaptation, total movement distance and vertical movement times of the rats in 4 min were observed and recorded (Pan et al., 2013). The splash test was carried out by spraying 10% sucrose solution on the back of animals in their home cage. The time spent grooming was recorded for 5 min after the open field test (OFT) was completed for 24 h (Lee et al., 2018). The SPT was used to evaluate anhedonia. Before testing, all rats were fasted and water-deprived for 24 h. On the day of the experiment, drinking water and 2% sucrose solution were placed in the home cage for 6 h. At the end of the experiment, the liquid content was measured and the sucrose preference was calculated with the following formula: Sucrose preference (%) = sucrose intake/(sucrose intake + water intake) × 100 (Shi et al., 2017). The NSFT was a conflict test in which food was removed from the cage 24 h before the experiment. During the experiment, the rats were placed in an open field (60 × 60 × 20 cm), and a small amount of food was placed in the center. The time of the first bite was recorded as the latent period of eating within 5 min. (David et al., 2009).

### Mechanism Detection

Enzyme-Linked Immunosorbent Assay Analysis for Interleukin 6, Tumor Necrosis Factor Alpha, Indoleamine 2,3-Dioxygenase, 5-Hydroxytryptamine, Brain-Derived Neurotrophic Factor and β-Nerve growth factor (β-NGF).

The levels of IL-6, TNF-α, indoleamine 2,3-dioxygenase (IDO), 5-hydroxytryptamine (5-HT), BDNF, and β-NGF were detected by enzyme-linked immunosorbent assay, according to the manufacturer’s instructions, and the optical density was measured at 450 nm by micro-plate reader.

### Total RNA Expression in Hippocampus and Cortex *via* Reverse Transcriptase Polymerase Chain Reaction

The total RNA was used to synthesize cDNA using the FastQuant RT kit (Tiangen, Beijing, China); subsequently, the amplification reactions were carried out in 96-well reaction plates with 20-µl reaction volume (Bio-Rad). The gene primer sequences of β-actin, IL-6, TNF-α, 5-HT1A, IDO1, BDNF, NGF, TrkB, TrkA, and CREB used in this study are listed in **Table 1**.

**Table 1:** Gene primer sequence.

Gene	Primer	Primer sequence (5′ to 3′)	Product size (bp)
β-actin	Forward Primer	CACCCGCGAGTACAACCTTC	207
	Reverse Primer	CCCATACCCACCATCACACC	
IL-6	Forward Primer	AGAGACTTCCAGCCAGTTGC	115
	Reverse Primer	CTGGTCTGTTGTGGGTGGTA	
TNF-α	Forward Primer	GATCGGTCCCAACAAGGAGG	138
	Reverse Primer	GCTTGGTGGTTTGCTACGAC	
5-HT1A	Forward Primer	TGATCTCGCTCACTTGGCTC	145
	Reverse Primer	AAAGCGCCGAAAGTGGAGTA	
IDO1	Forward Primer	GCATCAAGACCCGAAAGCAC	154
	Reverse Primer	GTTGCCCTTCCAACCAGACA	
BDNF	Forward Primer	TAGGCAGAATGAGCAATGTC	178
	Reverse Primer	CCCAAGAGGTAAAGTGTAGAAG	
NGF	Forward Primer	TGGAGATAAGACCACAGCCA	197
	Reverse Primer	TGACAAAGGTGTGAGTCGTG	
TrkB	Forward Primer	TGCTCAAGTTGGCGAGACAT	151
	Reverse Primer	GTCCCAGGAGTTCAGCTCAC	
TrkA	Forward Primer	CCCTCCTGATGTCTACGCCA	139
	Reverse Primer	CTCCTAGCCCAGAACGTCCA	
CREB	Forward Primer	AGCCGGGTACTACCATTC	244
	Reverse Primer	GCTGCTTCCCTGTTCTTC	

### Western Blotting Analysis

RIPA lysate buffer containing 1 mM Phenylmethanesulfonyl Fluoride (PMSF) was added to each sample to collect the total protein. The total protein concentration of each sample was determined by BCA method and adjusted all samples to the same concentration. The protein samples were mixed with a 5× loading buffer and denatured in at 95′C. The proteins were separated by Sodium Dodecylsulfonate- polyacrylamide gel electrophoresis (SDS-PAGE) (8% or 15%) and electrophoretically transferred onto polyvinylidene fluoride membranes. The membranes were probed with rabbit anti-TrkB antibody (1:1000; Cell Signaling Technology; Cat. No. #4603), rabbit anti-CREB antibody (1:1000; Cell Signaling Technology; Cat. No. #9197S), rabbit anti-p-CREB antibody (1:1000; Cell Signaling Technology; Cat. No. #9198S), rabbit anti-β-Tubulin antibody (1:1000; Cell Signaling Technology; Cat. No. #2128). Rabbit anti-brain-derived neurotrophic factor (BDNF) antibody (1:1000; Abcam; Cat. No. #ab108319). Rabbit anti-DLG4, PSD95-specific, polyclonal antibody (1:1000; Proteintech; Cat. No. #20665-1-AP) and rabbit anti-synaptophysin polyclonal antibody (1:1000; Proteintech; Cat. No. #17785-1-AP). Rabbit anti-GAPDH antibody (1:1000; Servicebio; Cat. No. #GB11002) overnight at 4′C and then incubated with Anti-rabbit IgG HRP-Linked Antibody (1:3000; Servicebio; Cat. No. #GB23303) at 37′C for 1.5 h. Detection was performed using a ChemiDoc XRS^+^ (BioRad, USA) image analysis system.

### Immunostaining

Brain tissue sections were deparaffinized and rehydrated (xylene I 15 min, xylene II 15 min, anhydrous ethanol I 5 min, anhydrous ethanol II 5 min, 85% ethanol 5 min, 75% ethanol 5 min, distilled water washing), and antigen repair was performed in a microwave oven (tissues were placed in a box filled with EDTA antigen repair buffer (pH 8.0), wash with phosphate-buffered saline (PBS) three times (5 min), treated with spontaneous fluorescence quenching agent 5 min, wash 10 min, blocked with bovine serum albumin for 30 min, sections were then incubated overnight with rabbit anti-IL-6 polyclonal antibody (1:300; Servicebio; Cat. No. #GB11117) at 4′C, wash with PBS and subsequently incubated with Alexa Fluor 488 goat anti-rabbit IgG (1:400; Servicebio; Cat. No. #GB25303) for 50 min at 37′C, wash with PBS three times (5 min), then the sections were incubated with DAPI for 10 min at room temperature to stain the cellular nuclei. A fluorescence microscope (Nikon Eclipse C1, Japan) was used to acquire images.

### Histological Analysis

Brain tissue sections were deparaffinized and rehydrated (xylene I 15 min, xylene II 15 min, then gradient alcohol dehydration: 100% I 5 min, 100% II 5 min, 95% 5 min, 90% 5 min, 80% 5 min, 70% 5 min, 50% 5 min), wash with PBS three times (5 min), and stained with 1% toluidine blue for 40 min, wash with PBS three times (5 min), and dehydrated in 70%, 80%, 95%, and 100% ethanol respectively, and then transparent with xylene, and the preparations were observed under microscope. The number of Nissl bodies in the brain tissues were calculated to evaluate the state of brain tissue.

### Statistical Analysis

All analyses were performed using SPSS. Data are presented as the mean ± SD. All analyses were performed using one-way ANOVA, *t*-test, or Mann–Whitney test rank sum analysis. The level of significance was set at *P* ≤ 0.05.

## Results

### Xiaoyao Pills Prevent an LPS-Induced Depressive Model

#### Xiaoyao Pills Prevent LPS-Induced Depressive Behavior

**Figure 2:**
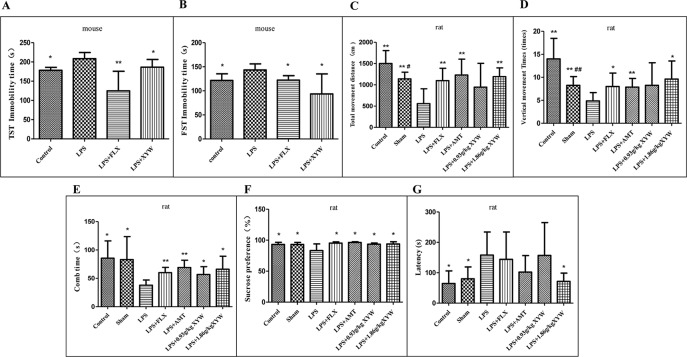
Lipopolysaccharide (LPS) administration acutely induced depression-like behavior. **(A)** Mice, the immobility time of the mice was prolonged in LPS group, compared with the control group (208.50 ± 15.90 vs 178.24 ± 7.62, N = 6 vs 5) (P 0.05), with 2 weeks of prior Xiaoyao Pills (XYW) administration (LPS + XYW), exhibited significantly lower immobility in the tail suspension test (208.50 ± 15.90 vs 186.38 ± 19.96, N = 6) (P 0.05) (Mann-Whitney test). **(B)** Mice, the immobility time of the mice was prolonged in LPS group, compared with the control group (143.39 ± 12.67 vs 121.55 ± 13.92, N = 6) (P 0.05), with 2 weeks of prior XYW administration (LPS + XYW), demonstrated significantly lower immobility in the forced swim test (143.39 ± 12.67 vs 93.56 ± 41.73, N = 6) (P 0.05) (t-test). **(C, D)** Rats, exposed to LPS exhibited significantly less distance travelled (558.75 ± 348.28 vs 1502.50 ± 303.73 (control) vs 1141.25 ± 156.79 (sham), N = 8) (P 0.05) and fewer number of vertical movements than the control and sham groups (4.88 ± 1.81 vs 14.00 ± 4.47 (control)vs 8.25 ± 1.91 (sham), N = 8) (P 0.05), with 2 weeks of prior XYW administration (LPS + XYW), exhibited a significantly higher distance travelled (558.75 ± 348.28 vs 945.00 ± 561.12 (0.93 g/kg) vs 1193.75 ± 204.80 (1.86 g/kg), N = 8) and times of vertical movement (4.88 ± 1.81 vs 8.25 ± 4.92 (0.93 g/kg) vs 9.63 ± 3.93 (1.86 g/kg), N = 8) (P 0.01) (t-test). **(E)** Rats, the LPS group exhibited a significantly lower comb time than the control and sham groups (37.88 ± 9.22 vs 85.50 ± 30.44 (control) vs 83.00 ± 40.62 (sham), N = 8) (P 0.05), with 2 weeks of prior XYW administration (LPS + XYW), exhibited a significantly longer time of combing (37.88 ± 9.22 vs 56.75 ± 13.77 (0.93 g/kg) vs 66.00 ± 22.90 (1.86 g/kg), N = 8) (n = 8, P 0.01) (Mann-Whitney test). **(F)** Rats, exposed to LPS exhibited a significantly lower saccharin preference than the control and sham groups [83.36 ± 10.72 vs 93.18 ± 3.44 (control) vs 93.25 ± 3.23 (sham), N = 8] (P 0.05), with 2 weeks of prior XYW administration (LPS + XYW), exhibited a significantly higher sucrose preference (83.36 ± 10.72 vs 93.62 ± 1.77 (0.93 g/kg) vs 93.91 ± 3.55 (1.86 g/kg), N = 8 vs 7 vs 8) (P 0.05) (t-test). **(G)** Rats, the LPS group exhibited a significantly longer latency to feed than the control and sham groups (158.00 ± 76.04 vs 64.63 ± 41.43 (control) vs 80.00 ± 39.14 (sham), N = 8) (P 0.05), with 2 weeks of prior XYW administration (LPS + XYW), exhibited a significantly shorter latency to feed (158.00 ± 76.04 vs 156.75 ± 108.30 (0.93 g/kg) vs 71.50 ± 27.40 (1.86 g/kg), N = 8) (n ≥ 7, P 0.05) (Mann-Whitney test). Compared with the control group, ^# ^*P* < 0.05,^## ^*P* < 0.01. Compared with the LPS group, *P < 0.05,**P < 0.01.

After intraperitoneal injection of LPS, the immobility time of the mice was prolonged in the TST and FST compared with the control group (*P* < 0.05), indicating that LPS treatment induced depression-related behavior. Mice administered XYW (483.6 mg·kg^-1^) for 2 weeks, by gavage, exhibited a significantly shorter immobility time during the TST and FST than the LPS group (*P* < 0.05) (**Figures 2A, B**).

In order to further study the effect of neuroinflammation on depressive model, the depression-like behaviors measured after LPS treatment are shown in (**Figures 2C–G**). In the OFT, rats exposed to LPS exhibited significantly less distance travelled (P < 0.05) and fewer number of vertical movements than the control and sham groups (P < 0.05), indicating that LPS treatment induced a decrease in locomotor activities and exploration. Rats administered with XYW (1.86 g·kg^-1^) for 2 weeks, by gavage, exhibited a significantly greater distance of total movement (P < 0.05) and number of vertical movements (P < 0.05) than the LPS group. In the splash test (ST), the LPS group exhibited a significantly lower comb time than the control and sham groups (P < 0.05). Rats administered with XYW (0.93, 1.86 g·kg^-1^) for 2 weeks displayed a significantly longer time of combing than the LPS group (P < 0.05). In the SPT, rats exposed to LPS exhibited a significantly lower saccharin preference than the control and sham groups (P < 0.05), indicating that LPS treatment induced anhedonia. Rats administered with XYW (0.93, 1.86 g·kg^-1^) for 2 weeks by gavage had a higher sucrose preference than the LPS group (P < 0.05). In the NSFT, the LPS group exhibited a significantly longer latency to feed than the control and sham groups, (P < 0.05), indicating that LPS treatment induced low desire for food in a novel environment. Rats administered for 2 weeks with XYW (1.86 g·kg^-1^) by gavage, displayed a decreased latency to feed than the LPS group (P < 0.05).

### Xiaoyao Pills Prevents LPS-Induced Inflammation

**Figure 3:**
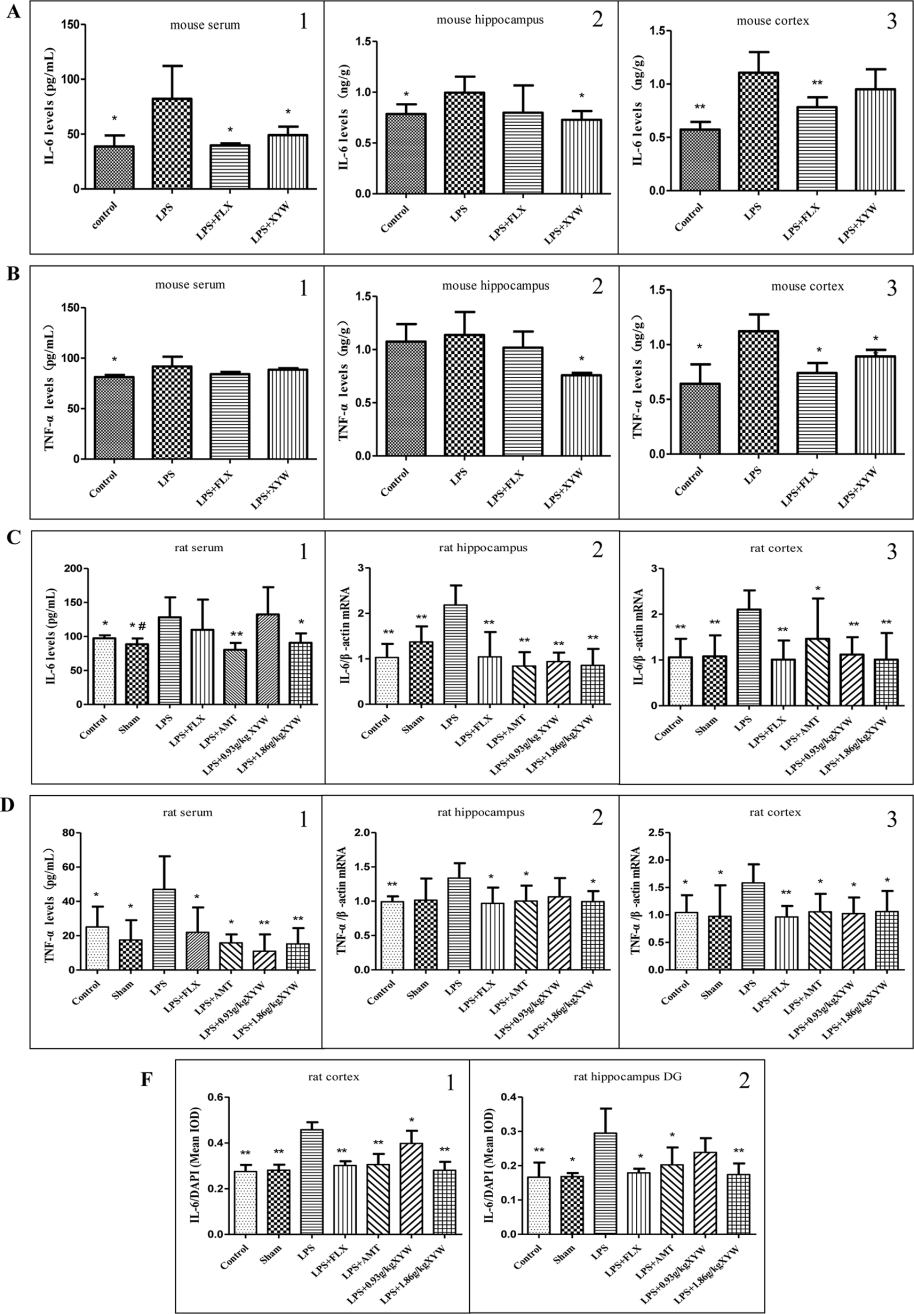
LPS administration acutely induced inflammation in different brain regions. **(A, B)** Mice, LPS administration increased levels of cytokines including IL-6 (serum: 82.23 ± 29.99 vs 38.73 ± 10.10, N = 6 vs 5, cortex: 1.11 ± 0.19 vs 0.57 ± 0.07, N = 5, hippocampus: 1.00 ± 0.16 vs 0.79 ± 0.10, N = 5) and TNF-α (serum: 91.84 ± 9.60 vs 81.31 ± 2.13, N = 6 vs 5, cortex: 1.12 ± 0.15 vs 0.64 ± 0.18, N = 4, hippocampus: 1.14 ± 0.22 vs 1.07 ± 0.16, N = 5) in the serum and cortex (P 0.05), and the level of IL-6 in the hippocampus. with 2 weeks of prior XYW administration (LPS + XYW), exhibited significantly lower levels of Interleukin 6 (IL-6) (serum: 82.23 ± 29.99 vs 49.01 ± 7.78, N = 6, cortex: 1.11 ± 0.19 vs 0.95 ± 0.19, N = 5, hippocampus: 1.00 ± 0.16 vs 0.73 ± 0.09, N = 5) and Tumor necrosis factor alpha (TNF-α) [serum: 91.84 ± 9.60 vs 88.51 ± 1.51, N = 6 vs 5, cortex: 1.12 ± 0.15 vs 0.89 ± 0.06, N = 4, hippocampus:1.14 ± 0.22 vs 0.76 ± 0.02, N = 5] in different regions (P 0.05) (A1-2, B1-2: t-test, A3, B3: Mann-Whitney test). **(C, F)** Rats, LPS increased levels of IL-6 (serum:128.18 ± 29.32 vs 97.34 ± 4.38 (control) vs 88.60 ± 8.53 (sham), N = 8 vs 6 vs 6, cortex:2.10 ± 0.42 vs 1.06 ± 0.41 (control) vs 1.08 ± 0.46 (sham), N = 6, hippocampus: 2.18 ± 0.43 vs 1.03 ± 0.30 (control) vs 1.37 ± 0.34 (sham), N = 6 vs 5 vs 5, cortex (IL-6/DAPI): 0.46 ± 0.03 vs 0.27 ± 0.03 (control) vs 0.28 ± 0.02 (sham), N = 4 vs 5 vs 5, hippocampus (IL-6/DAPI): 0.29 ± 0.07 vs 0.17 ± 0.04 (control) vs 0.17 ± 0.01 (sham), N = 5 vs 4 vs 5) and TNF-α [serum: 47.03 ± 19.28 vs 25.12 ± 11.84 (control) vs 17.49 ± 11.53 (sham), N = 7 vs 8 vs 7, cortex:1.58 ± 0.34 vs 1.04 ± 0.31 (control) vs 0.97 ± 0.57 (sham), N = 6, hippocampus:1.34 ± 0.22 vs 0.99 ± 0.08 (control) vs 1.02 ± 0.31 (sham), N = 6 vs 5 vs 5] (P 0.05 or P 0.01), with 2 weeks of prior XYW administration (LPS + XYW), exhibited significantly lower levels of cytokines including IL-6 (serum: 128.18 ± 29.32 vs 132.40 ± 40.05 (0.93 g/kg) vs 90.73 ± 13.78 (1.86 g/kg), N = 8, cortex: 2.10 ± 0.42 vs 1.12 ± 0.38 (0.93 g/kg) vs 1.01 ± 0.58 (1.86 g/kg), N = 6, hippocampus:2.18 ± 0.43 vs 0.94 ± 0.20 (0.93 g/kg) vs 0.86 ± 0.36 (1.86 g/kg), N = 6 vs 5 vs 6, cortex (IL-6/DAPI): 0.46 ± 0.03 vs 0.40 ± 0.05 (0.93 g/kg) vs 0.28 ± 0.04 (1.86 g/kg), N = 4 vs 5 vs 5, hippocampus (IL-6/DAPI): 0.29 ± 0.07 vs 0.24 ± 0.04 (0.93 g/kg) vs 0.17 ± 0.03 (1.86 g/kg), N = 5) and TNF-α [serum:47.03 ± 19.28 vs 10.94 ± 9.81 (0.93 g/kg) vs 15.26 ± 9.17 (1.86 g/kg), N = 7, cortex: 1.58 ± 0.34 vs 1.02 ± 0.29 (0.93 g/kg) vs 1.06 ± 0.37 (1.86 g/kg), N = 6, hippocampus: 1.34 ± 0.22 vs 1.06 ± 0.27 (0.93 g/kg) vs 0.99 ± 0.15 (1.86 g/kg), N = 6 vs 5 vs 6] in peripheral and central brain regions (P 0.05 or P 0.01). (C1, D1: Mann-Whitney test, C2, D2-3, F2: t-test, C3, F1: one-way ANOVA). Compared with the control group, ^#^ *P* < 0.05, ^## ^*P* < 0.01. Compared with the LPS group, *P < 0.05,**P < 0.01.

LPS administration by intraperitoneal injection induced inflammation in different brain regions, with increased levels of cytokines including IL-6 and TNF-α in the serum and cortex (P < 0.05), and the level of IL-6 in the hippocampus. Pre-treatment with Xiaoyao Pills (483.6 mg·kg^-1^) blocked the increase in the level of IL-6 in the serum and hippocampus (P < 0.05), but not in the cortex (**Figure 3A**). Interestingly, in the hippocampus and cortex, we detected decreased levels of TNF-α (P < 0.05), but not in serum (**Figure 3B**).

Similar to the results in mice, the pro-inflammatory cytokine-inducer LPS was infused intracerebroventricularly, which induced neuroinflammation with increased levels of cytokines including IL-6 and TNF-α (**Figures 3C–F**). The results between peripheral and central cytokine levels suggests that Xiaoyao Pills might inhibit inflammation in rats. The LPS treated group showed an increased secretion of IL-6 and TNF-α in the serum (P < 0.05). Rats administered with XYW (1.86 g·kg^-1^) for 2 weeks by gavage displayed lower levels of IL-6 and TNF-α in serum than the LPS group (P < 0.05). Treatment with Xiaoyao Pills (0.93 g·kg^-1^) downregulated the levels of TNF-α in serum (P < 0.05). We therefore examined mRNA levels of the two cytokines, using reverse transcriptase polymerase chain reaction, in the cortex and hippocampus. We found that the transcription levels of IL-6 and TNF-α were upregulated in the cortex and hippocampus following LPS administration, whereas, rats administered with XYW (1.86 g·kg^-1^) for 2 weeks by gavage, displayed a lower expression of IL-6 and TNF-α in the cortex and hippocampus than the model group (P < 0.05). Treatment with Xiaoyao Pills (0.93 g·kg^-1^) also downregulated the transcription levels of IL-6 in the cortex and hippocampus (P < 0.05) and TNF-α in the cortex (P < 0.05). We further examined the expression of IL-6 in the brain, using immunofluorescent staining, which demonstrated the protein levels of IL-6 in the cortex and hippocampus were higher in the LPS treatment group than the control (P < 0.05). We found that in rats administered with XYW (1.86 g·kg^-1^, 0.93 g·kg^-1^) 2 weeks displayed a lower expression of IL-6 in the cortex and hippocampus (P < 0.05). These data show that pre-treatment with Xiaoyao Pills decreases local cytokine production in the cortex and hippocampus.

### Xiaoyao Pills Prevents LPS-Induced Limited 5-HT and IDO

**Figure 4:**
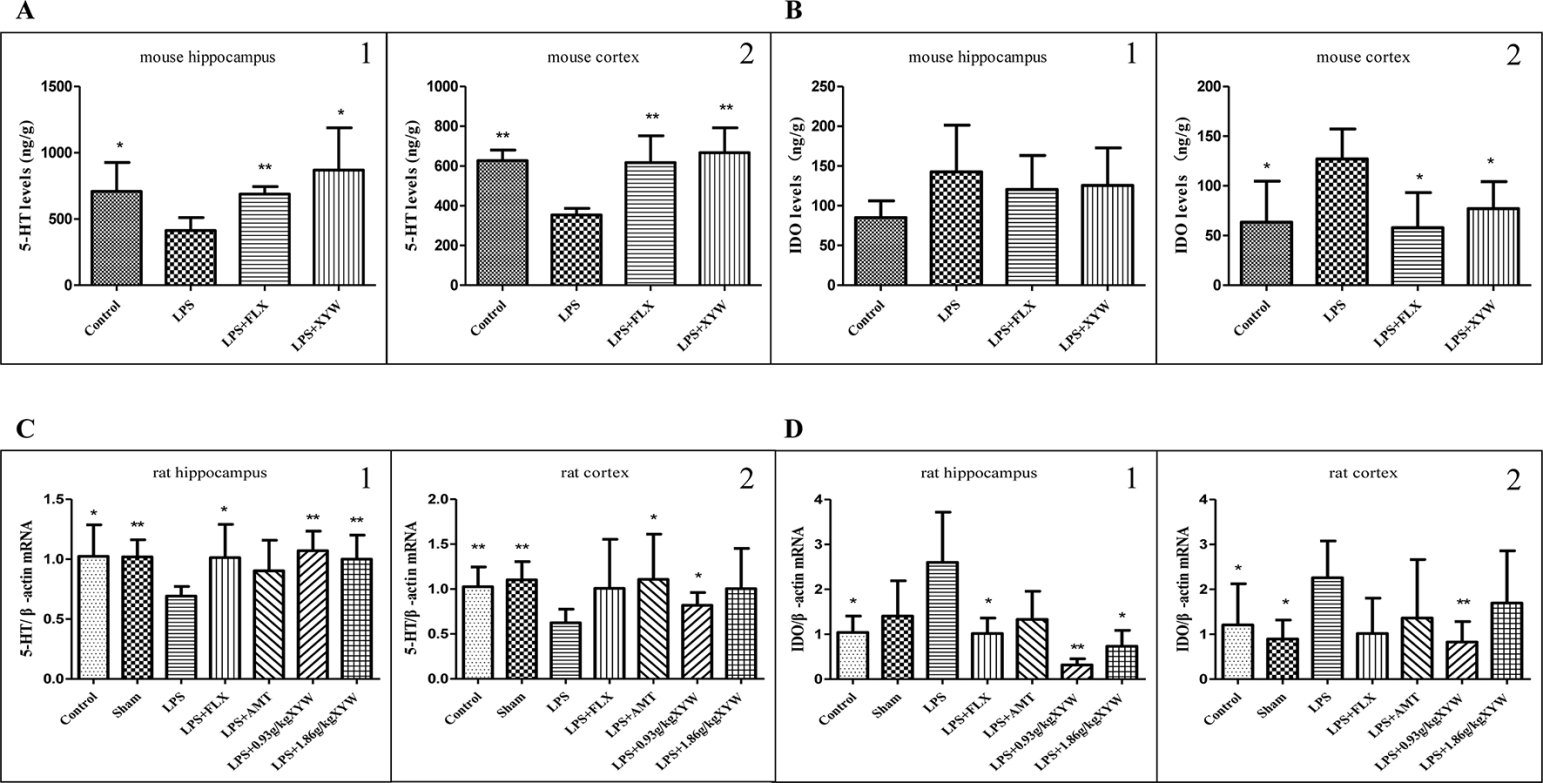
LPS administration acutely induced inflammation in different brain regions. **(A)** Mice, LPS administration resulted in significantly lower levels of 5-HT in the hippocampus and cortex than in control groups (cortex:35.40 ± 3.29 vs 62.70 ± 5.36, N = 5, hippocampus:41.27 ± 9.84 vs 70.85 ± 21.88, N = 5) (P 0.05 or P 0.01). with 2 weeks of prior XYW administration (LPS + XYW), exhibited significantly higher levels of 5-hydroxytryptamine (5-HT) in different regions (cortex: 35.40 ± 3.29 vs 66.67 ± 12.55, N = 5, hippocampus: 41.27 ± 9.84 vs 86.91 ± 31.91, N = 5) (P 0.05 or P 0.01) (A1-2: t-test). **(B)** Mice, LPS administration resulted in significantly increased level of IDO in cortex than in control groups (cortex:12.71 ± 3.01 vs 6.35 ± 4.13, N = 5, hippocampus: 14.25 ± 5.88 vs 8.52 ± 2.09, N = 5) (P 0.05). with 2 weeks of prior XYW administration (LPS + XYW), demonstrated significantly lower levels of Indoleamine 2,3-dioxygenase (IDO) in different regions (cortex: 12.71 ± 3.01 vs 7.71 ± 2.72, N = 5, hippocampus: 14.25 ± 5.88 vs 12.56 ± 4.73, N = 5) (P 0.05 or P 0.01) (B1-2: t-test). **(C, D)** Rats, LPS-induced decreased in 5-HT transcription (cortex:0.62 ± 0.15 vs 1.03 ± 0.22 (control) vs 1.10 ± 0.20 (sham), N = 6, hippocampus: 0.69 ± 0.08 vs 1.02 ± 0.26 (control) vs 1.02 ± 0.14 (sham), N = 6 vs 5 vs 5) and increase in IDO transcription in the cortex and hippocampus [cortex:2.26 ± 0.82 vs 1.21 ± 0.9 (control) vs 0.89 ± 0.42 (sham), N = 6, hippocampus: 2.60 ± 1.11 vs 1.04 ± 0.36 (control) vs 1.40 ± 0.79 (sham), N = 6 vs 5 vs 5] (P 0.05 or P 0.01), with 2 weeks of prior XYW administration (LPS + XYW), exhibited significantly higher levels of 5-hydroxytryptamine (5-HT) [cortex: 0.62 ± 0.15 vs 0.82 ± 0.14 (0.93 g/kg) vs 1.00 ± 0.45 (1.86 g/kg), N = 6, hippocampus: 0.69 ± 0.08 vs 1.07 ± 0.16 (0.93 g/kg) vs 1.00 ± 0.20 (1.86 g/kg), N = 6 vs 5 vs 6] and lower levels of Indoleamine 2,3-dioxygenase (IDO) in the cortex and hippocampus [cortex::2.26 ± 0.82 vs 0.83 ± 0.46 (0.93 g/kg) vs 1.70 ± 1.16 (1.86 g/kg), N = 6, hippocampus: 2.60 ± 1.11 vs 0.32 ± 0.14 (0.93 g/kg) vs 0.73 ± 0.35 (1.86 g/kg), N = 6 vs 5 vs 6] (P 0.05 or P 0.01) (C1-2: t-test, D1-2: Mann-Whitney test). Compared with the LPS group, *P < 0.05,**P < 0.01.

LPS administration resulted in significantly lower levels of 5-HT in the hippocampus and cortex, and significantly increased level of IDO in cortex than in control and sham groups (P < 0.05). Mice administered XYW (483.6 mg·kg^-1^) for 2 weeks had higher 5-HT levels in the hippocampus and cortex than the model (P < 0.05) (**Figure 4A**). Also, pre-treatment with Xiaoyao Pills (483.6 mg·kg^-1^) resulted in lower levels of IDO in the cortex than the LPS group (P < 0.05) (**Figure 4B**).

It is known that LPS-induced depression depends on activation of IDO signaling pathway (OConnor et al., 2009). We detected an LPS-induced increase in IDO transcription in the cortex and hippocampus (P < 0.05), which was prevented by pre-treatment with Xiaoyao Pills (**Figures 4C, D**). Rats administered XYW (0.93 g·kg^-1^) by gavage had lower transcription levels of IDO in the cortex and hippocampus (P < 0.05) and higher transcription levels of 5-HT in the cortex and hippocampus than the LPS group (P < 0.05). Xiaoyao Pills (1.86 g·kg^-1^) decreased the expression of IDO in the hippocampus (P < 0.05), and increased the transcription level of 5-HT in the hippocampus (P < 0.05).

### Xiaoyao Pills Prevents LPS-Induced Reduction of Neurotrophic Factor

**Figure 5:**
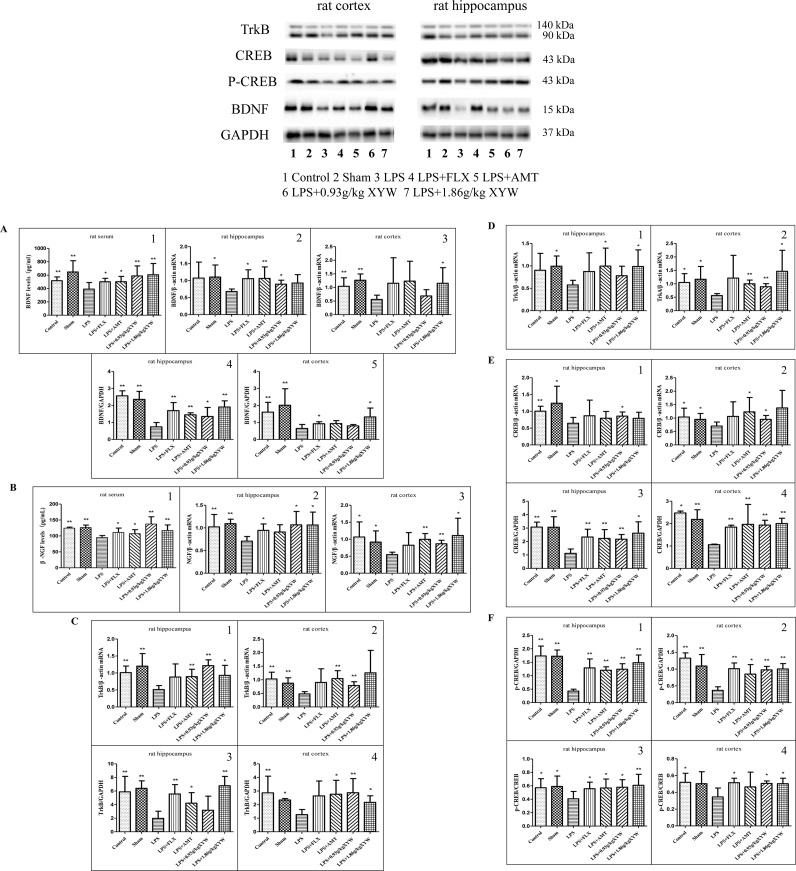
LPS administration acutely reduced the expression of brain-derived neurotrophic factor (BDNF)/neuronal growth factor (NGF)-Tropomyosin receptor kinase (Trk) B/TrkA- cAMP response element-binding protein (CREB) in different brain regions. **(A, F)** Rats, The expression levels of BDNF/NGF-TrkB/TrkA-CREB following LPS treatment were significantly lower than those in the control and sham groups (P 0.05 or P 0.01), with 2 weeks of prior XYW administration (LPS + XYW [0.93,1.86 g·kg^-1^]), exhibited significantly higher levels of BDNF and NGF in serum, and Xiaoyao Pills (1.86 g·kg^-1^) caused higher transcription levels of BDNF in the cortex, NGF and TrkA in the cortex and hippocampus and TrkB in the hippocampus. Xiaoyao Pills (0.93 g·kg^-1^) treatment resulted in a higher transcription level of BDNF in the hippocampus, NGF, TrkB and CREB in the cortex and hippocampus, and TrkA in the cortex. Xiaoyao Pills (1.86 g·kg^-1^) pre-treatment could add the translation levels of BDNF, TrkB, p-CREB, CREB and increased the ratio of p-CREB/CREB in the cortex and hippocampus. The Xiaoyao Pills (0.93 g·kg^-1^) group had a higher level of translation of BDNF in the hippocampus, TrkB in the cortex, p-CREB, CREB in the cortex and hippocampus, and a higher p-CREB/CREB ratio in the cortex and hippocampus (n≥5, P 0.05 or P 0.01) (A1, A3, A5, B1, B3, C1, D1-2, E1-2, F4: t-test, A2, B2, C2, C4, E3-4, F2: Mann-Whitney test, A4, C3, F1, F3: one-way ANOVA). BDNF: (serum:390.23 ± 98.45 vs 516.18 ± 56.72 (control) vs 646.87 ± 168.63 (sham) vs 587.51 ± 150.23 (0.93 g/kg) vs 605.59 ± 168.54 (1.86 g/kg), N = 8, cortex (PCR): 0.56 ± 0.16 vs 1.04 ± 0.31 (control) vs 1.27 ± 0.23 (sham) vs 0.69 ± 0.23 (0.93 g/kg) vs 1.15 ± 0.58 (1.86 g/kg), N = 6, hippocampus (PCR):0.68 ± 0.07 vs 1.08 ± 0.47 (control) vs 1.11 ± 0.36 (sham) vs 0.90 ± 0.12 (0.93 g/kg) vs 0.93 ± 0.25 (1.86 g/kg), N = 6 vs 5 vs 5 vs 5 vs 6, cortex (Western blot):0.65 ± 0.24 vs 1.61 ± 0.58 (control) vs 2.01 ± 0.97 (sham) vs 0.80 ± 0.08 (0.93 g/kg) vs 1.32 ± 0.52 (1.86 g/kg), N = 6 vs 6 vs 6 vs 5 vs 6, hippocampus (Western blot): 0.74 ± 0.25 vs 2.57 ± 0.29 (control) vs 2.35 ± 0.47 (sham) vs 1.35 ± 0.53 (0.93 g/kg) vs 1.91 ± 0.36 (1.86 g/kg), N = 6). NGF: [serum:95.09 ± 6.37 vs 124.38 ± 3.12 (control) vs 126.26 ± 7.85 (sham) vs 137.44 ± 22.72 (0.93 g/kg) vs 117.26 ± 17.36 (1.86 g/kg), N = 8 vs 7 vs 7 vs 7 vs 8, cortex:0.55 ± 0.72 vs 1.06 ± 0.44 (control) vs 0.92 ± 0.33 (sham) vs 0.87 ± 0.10 (0.93 g/kg) vs 1.11 ± 0.51 (1.86 g/kg), N = 6, hippocampus:0.71 ± 0.10 vs 1.02 ± 0.27 (control) vs 1.09 ± 0.10 (sham) vs 1.07 ± 0.29 (0.93 g/kg) vs 1.06 ± 0.28 (1.86 g/kg), N = 6 vs 5 vs 5 vs 5 vs 6]. TrkB: (PCR: cortex: 0.48 ± 0.08 vs 1.03 ± 0.25 (control) vs 0.87 ± 0.20 (sham) vs 0.79 ± 0.14 (0.93 g/kg) vs 1.26 ± 0.83 (1.86 g/kg), N = 6, hippocampus: 0.51 ± 0.11 vs 1.01 ± 0.19 (control) vs 1.20 ± 0.37 (sham) vs 1.22 ± 0.17 (0.93 g/kg) vs 0.93 ± 0.29 (1.86 g/kg), N = 5 vs 5 vs 5 vs5 vs 6). [Western blot: cortex: 1.27 ± 0.37 vs 2.87 ± 1.23 (control) vs 2.34 ± 0.11 (sham) vs 2.87 ± 1.04 (0.93 g/kg) vs 2.17 ± 0.48 (1.86 g/kg), N = 6 vs 6 vs 4 vs 6 vs 5, hippocampus: 1.98 ± 1.05 vs 5.88 ± 2.28 (control) vs 6.39 ± 1.10 (sham) vs 3.17 ± 2.07 (0.93 g/kg) vs 6.78 ± 1.36 (1.86 g/kg), N = 6]. TrkA: [PCR: cortex: 0.56 ± 0.07 vs 1.05 ± 0.33 (control) vs 1.17 ± 0.47 (sham) vs 0.89 ± 0.11 (0.93 g/kg) vs 1.46 ± 0.78 (1.86 g/kg), N = 6, hippocampus: 0.57 ± 0.10 vs 0.90 ± 0.38 (control) vs 0.99 ± 0.23 (sham) vs 0.78 ± 0.21 (0.93 g/kg) vs 0.99 ± 0.37 (1.86 g/kg), N = 6 vs 5 vs 5 vs 5 vs 6]. CREB: [PCR: cortex: 0.70 ± 0.15 vs 1.04 ± 0.33 (control) vs 0.95 ± 0.22 (sham) vs 0.95 ± 0.15 (0.93 g/kg) vs 1.38 ± 0.65 (1.86 g/kg), N = 6, hippocampus: 0.65 ± 0.17 vs 1.00 ± 0.15 (control) vs 1.24 ± 0.50 (sham) vs 0.86 ± 0.12 (0.93 g/kg) vs 0.79 ± 0.18 (1.86 g/kg), N = 6 vs 5 vs 5 vs 5 vs 6]. [Western blot: cortex: 1.05 ± 0.03 vs 2.47 ± 0.09 (control) vs 2.19 ± 0.43 (sham) vs 1.93 ± 0.22 (0.93 g/kg) vs 2.00 ± 0.24 (1.86 g/kg), N = 5 vs 4 vs 6 vs 6 vs 6, hippocampus: 1.12 ± 0.32 vs 3.07 ± 0.37 (control) vs 3.06 ± 0.78 (sham) vs 2.17 ± 0.35 (0.93 g/kg) vs 2.62 ± 0.84 (1.86 g/kg), N = 6]. p-CREB: [Western blot: cortex: 0.37 ± 0.11 vs 1.33 ± 0.16 (control) vs 1.09 ± 0.34 (sham) vs 0.98 ± 0.10 (0.93 g/kg) vs 1.01 ± 0.16 (1.86 g/kg), N = 6, hippocampus: 0.43 ± 0.07 vs 1.74 ± 0.37 (control) vs 1.73 ± 0.23 (sham) vs 1.24 ± 0.21 (0.93 g/kg) vs 1.48 ± 0.29 (1.86 g/kg), N = 6]. p-CREB/CREB: [Western blot: cortex: 0.34 ± 0.11 vs 0.52 ± 0.11 (control) vs 0.50 ± 0.14 (sham) vs 0.51 ± 0.03 (0.93 g/kg) vs 0.50 ± 0.07 (1.86 g/kg), N = 5 vs 6 vs 6 vs 6 vs 6, hippocampus: 0.41 ± 0.11 vs 0.57 ± 0.13 (control) vs 0.59 ± 0.15 (sham) vs 0.58 ± 0.11 (0.93 g/kg) vs 0.61 ± 0.16 (1.86 g/kg), N = 6].

BDNF signaling is required for both migration and survival of neurons. BDNF-TrkB-CREB signaling was also affected by acute LPS administration, and pre-treatment with Xiaoyao Pills ameliorated the downregulation of neurotrophic factor (Marshall et al., 2018). The expression levels of BDNF/NGF-TrkB/TrkA-CREB following LPS treatment were significantly lower than those in the control and sham groups (P < 0.05) (**Figures 5A–F**). We found that this pathway was activated when rats were administered XYW for 2 weeks by gavage. Xiaoyao Pills (0.93, 1.86 g·kg^-1^) could increase BDNF and NGF levels in serum (P < 0.01), and Xiaoyao Pills (1.86 g·kg^-1^) could increase the transcription level of BDNF in the cortex, NGF and TrkA in the cortex and hippocampus, and TrkB in the hippocampus (P < 0.05 or P < 0.01). Xiaoyao Pills (0.93 g·kg^-1^) increased the transcription level of BDNF in the hippocampus, NGF, TrkB and CREB in both the cortex and the hippocampus, and TrkA in the cortex (P < 0.05). We then investigated the levels of translation of BDNF, TrkB, CREB and p-CREB. The translation levels of BDNF, TrkB, p-CREB and CREB were significantly higher in the Xiaoyao Pills (1.86 g·kg^-1^) pre-treatment group than the model group, as was the ratio of p-CREB/CREB in the cortex and hippocampus (P < 0.05). Pre-treatment with Xiaoyao Pills (1.86 g·kg^-1^) increased the translation levels of BDNF, TrkB, p-CREB, CREB and elevation the ratio of p-CREB/CREB in cortex and hippocampus than in the LPS group (P < 0.05), Xiaoyao Pills (0.93 g·kg^-1^) also promoted the translation levels of BDNF in hippocampus, TrkB in cortex, p-CREB, CREB in cortex and hippocampus, and increase p-CREB/CREB in cortex and hippocampus (P < 0.05).

### Xiaoyao Pills Promotes LPS-Damaged Synaptic Growth

**Figure 6:**
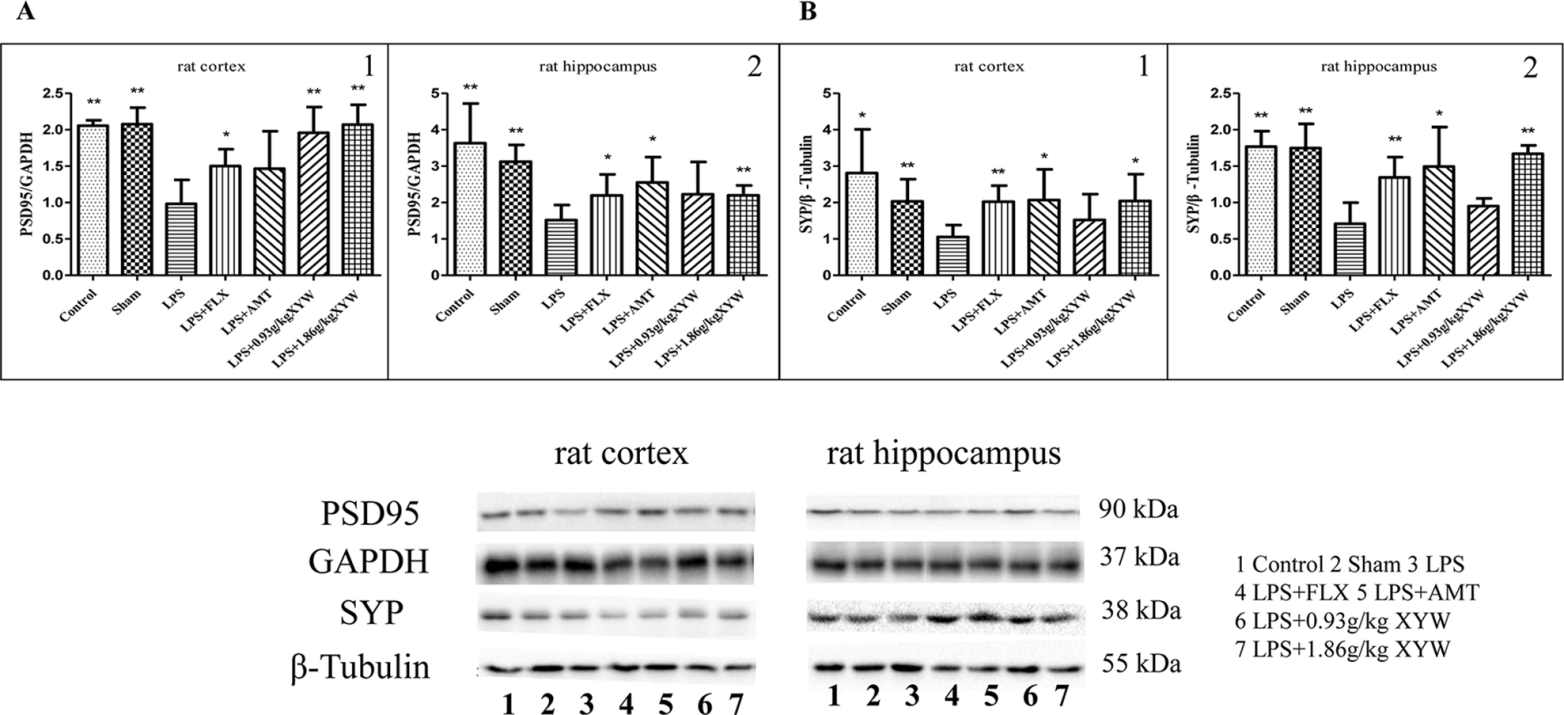
LPS administration acutely reduced postsynaptic density protein 95 (PSD)-95 and synaptophysin (SYP) in different brain regions. **(A, B)** Rats, PSD95 (cortex:0.98 ± 0.33 vs 2.05 ± 0.08 (control) vs 2.08 ± 0.23 (sham), N = 6, hippocampus: 1.52 ± 0.41 vs 3.63 ± 1.09 (control) vs 3.12 ± 0.46 (sham), N = 6) and SYP (cortex:1.05 ± 0.33 vs 2.81 ± 1.20 (control) vs 2.03 ± 0.61 (sham), N = 6, hippocampus: 0.71 ± 0.29 vs 1.77 ± 0.21 (control) vs 1.75 ± 0.33 (sham), N = 6) were lower in the hippocampus and cortex in LPS treated group than the control (P 0.05 or P 0.01). with 2 weeks of prior XYW administration (LPS + XYW [1.86 g·kg^-1^]), exhibited a significantly higher level of PSD-95 and SYP in the cortex and hippocampus. 0.93 g·kg^-1^ Xiaoyao Pills also increased PSD-95 protein in the cortex (PSD-95: cortex:0.98 ± 0.33 vs 1.96 ± 0.35 (0.93 g/kg) vs 2.07 ± 0.27 (1.86 g/kg), N = 6, hippocampus: 1.52 ± 0.41 vs 2.23 ± 0.89 (0.93 g/kg) vs 2.20 ± 0.27 (1.86 g/kg), N = 6) (SYP: cortex: 1.05 ± 0.33 vs 1.52 ± 0.71 (0.93 g/kg) vs 2.04 ± 0.73 (1.86 g/kg), N = 6, hippocampus: 0.71 ± 0.29 vs 0.95 ± 0.11 (0.93 g/kg) vs 1.67 ± 0.12 (1.86 g/kg), N = 6) (P 0.05 or P 0.01) (A1: Mann-Whitney test, A2, B1-2: t-test). Compared with the LPS group, *P < 0.05,**P < 0.01.

Alterations in synaptic proteins may be a major cause of learning and memory impairment (Zhang et al., 2016). To further analyses the protective effect of Xiaoyao Pills against LPS, we assessed synaptic protein markers. As expected, expression of PSD95 and SYP were lower in the hippocampus and cortex in LPS treated group than the control (**Figures 6A, B**). However, PSD-95 and SYP protein levels were higher with Xiaoyao Pills (1.86 g·kg^-1^) treatment in the hippocampus and cortex (P < 0.05). Xiaoyao Pills (0.93 g·kg^-1^) also increased PSD-95 protein in the cortex (P < 0.05). These results suggest that the neuroprotective function of Xiaoyao Pills in LPS-induced synaptotoxicity may occur by increasing the expression of synaptic proteins.

### Xiaoyao Pills Prevent LPS-Induced Injury to Nissl Bodies in Hippocampal CA3 Area

**Figure 7:**
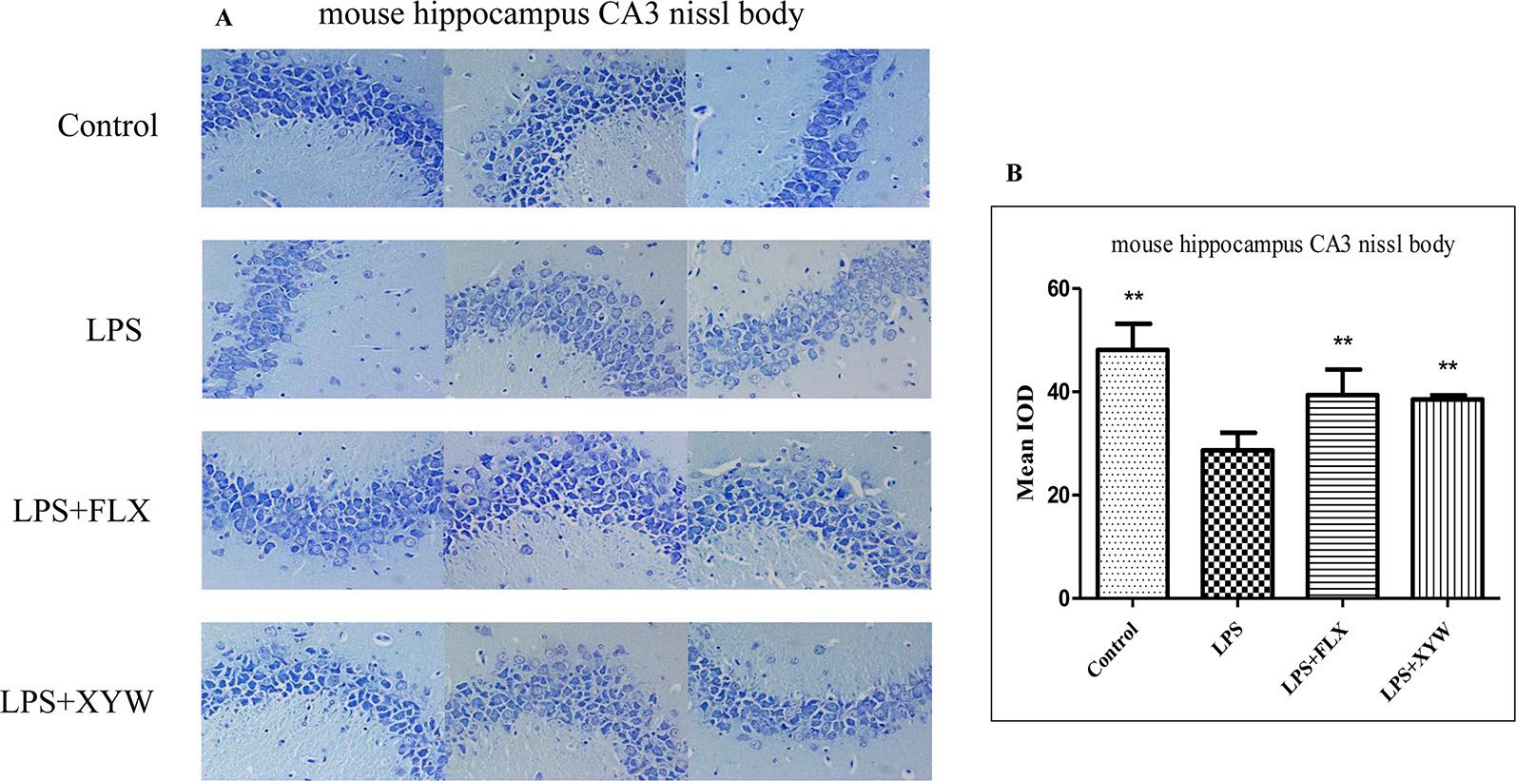
LPS administration caused hippocampal neuronal damage. **(A, B)** Mice, LPS-treated reduced Nissl bodies, and a significant decrease in the average optical density [28.69 ± 3.40 vs 48.14 ± 5.03 (control), N = 4] (P 0.05 or P 0.01), with 2 weeks of prior XYW administration (LPS + XYW), exhibited a significant improvement of the average optical density value in Nissl bodies than the control rats (28.69 ± 3.40 vs 38.54 ± 0.78, N = 4) (P 0.01) (B: t-test).

Nissl staining showed that hippocampal neurons in the LPS-treated mouse model were damaged, and manifested irregular arrangement, unclear stratification, reduced Nissl bodies, and a significant decrease in the average optical density (P < 0.05, **Figure 7A**). The average optical density of the XYW group was significantly higher than that of the model group (P < 0.05, **Figure 7B**). This result showed that XYW could improve hippocampal neuronal damage cause by LPS.

## Discussion

Xiaoyao San is a prescription for Xiaoyao Pills and is included in the Chinese Pharmacopoeia. They are prepared from Xiaoyao San Decoction, and are more convenient to take, with equal efficacy. According to the theory of traditional Chinese Medicine, Xiaoyao San has been most frequently used to smooth the liver, strengthen the spleen, and nourish the blood. And Xiaoyao San has complex composition and many targets for exerting effects, the treatment of depression is one of its applications. The dosage setting of traditional Chinese medicine prescription is adjusted according to the severe degree of symptoms, which usually within a range. So the reference dose is not targeted. Thus, our laboratory made appropriate adjustments based on previous studies. The highest dose of Xiaoyao Pills in this study is 1.86 g·kg^-1^ per day that is the best dose for Xiaoyao Pills to exert antidepressant effects in rodents. And more importantly, this dose did not show toxic effects.

The results demonstrated that LPS is a pro-inflammation mediator which can evoke inflammation in the serum, hippocampus and cortex of animals. This was defined by elevated levels of pro-inflammatory cytokines and the release of various inflammation-related mediators, including IL-6, TNF-α and IDO. This neuroinflammatory state causes significant depressive-like behaviors in the animals, including reduced locomotor activity in the OFT, reduced saccharin preference, added immobility time in the TST and FST, decreased comb time in ST and increased latency to food in the NSFT. These neuroinflammatory responses also inhibited nerve growth and decreased Nissl bodies, which was marked by decreased levels of neurotrophic factor and related factors, synaptic proteins, and pathomorphology.

The LPS-induced animal model of depression is widely used in behavioral tests such as the FST, TST, SPT, ST, SPT, and NSFT for evaluating the efficacy of chronic antidepressant treatments. The FST and TST are used for assessing “behavioral despair”, resulting in a reduction of locomotor activity which is linked to reductions in energy generation (Okan and Gundeniz, 2010). The SPT serves as an index of anhedonia-like behavior, while the NSFT is a conflict test that triggers competitive motivation (David et al., 2009). In this study an increase in immobility time during the FST and TST was interpreted as an indicator of depression. Our results showed that LPS exposure caused significant increases in immobility times. Pre-treatment with Xiaoyao Pills dramatically decreased immobility times of depressive rats. Similarly, in the OFT, LPS exposure significantly reduced the distance of total movement and times of vertical movement, suggesting a loss of exploration and interest to a novel environment. This reduction in locomotor activity was also ameliorated by pre-treatment with Xiaoyao Pills. The ST, used as an index of self-care and motivational behavior, which are considered to reflect some symptoms of depression such as apathetic behavior, in this test, the LPS treated group exhibited a significant decrease in comb time. Xiaoyao Pills increased the time of combing, and rats subjected to LPS consumed lower amounts of sucrose solution than the control or sham group. The NSFT revealed that LPS treatment induced low desire for food in a novel environment (Katz et al., 1981; Willner, 2005). Pre-treatment with Xiaoyao Pills prevented this behavioral change and reduced latency to feed. Taken together, these behavioral results suggest that Xiaoyao Pills treatment exerts antidepressant-like effects in this LPS-induced animal model of depression.

Growing evidence suggests that activation of the immune response often results in neuroinflammatory responses and consequently induces neuropsychiatric symptoms in animal models and humans (Jangra et al., 2016, Zhao et al., 2019). Specifically, inflammation occurs in the nervous system, termed “neuroinflammation,” which is typically associated with the production of cytokines. Pro-inflammatory cytokines are secreted, including IL-6 and TNF-α. A recent study has been reported that the cytokines might be used as endogenous biomarkers to monitor the efficacy of antidepressants (Clarke et al., 2017). IDO is a pivotal metabolic enzyme in inflammation-induced neuroimmune responses that is activated by inflammatory cytokines (i.e. IL-6, TNFα). It is the rate-limiting enzyme that metabolizes the essential amino acid tryptophan along the kynurenine pathway. Tryptophan is the biosynthetic precursor of the neurotransmitter serotonin (5-HT) that is well known to be important in the regulation of mood. The increased degradation of tryptophan along the kynurenine pathway could potentially deplete the bioavailability of this amino acid precursor for serotonin synthesis. Accordingly, the increased activity of IDO has been implicated as a critical molecular mediator of inflammation-induced depressive-like behavior (Da et al., 2016). In the present study, the LPS-induced depression model produced the proinflammatory cytokines (IL-6 and TNF-α), and IL-6 positive cells were identified primarily in the dentate gyrus (DG) of rats. Additionally, the expression of IDO was increased, while the level of 5-HT was decreased. These indicate that LPS generates a reliable and useful model of neuroinflammation-induced depression (O’Connor et al., 2009), however, in this study, treatment with Xiaoyao Pills ameliorated the effects of LPS treatment.

Neurotrophic factors are important signaling molecules in the brain, responsible for axon localization, neuronal growth, synaptic maturation and synaptic plasticity during development. This family of molecules, including NGF and BDNF, are prevalent growth factors in the central nervous system. They are implicated in psychiatric diseases such as depression. Mature BDNF signals through its high-affinity receptor, TrkB. When BDNF is bound to TrkB, the latter is dimerized and autophosphorylated. This leads to activation of intracellular signaling cascades, which can activate the transcription factor CREB (Autry and Monteggia, 2012). CREB, a key nuclear transcription factor, activation promotes the transcription and translation of BDNF. It has been demonstrated that chronic stress leads to a reduction in hippocampal BDNF levels in rats (Nibuya et al., 1995). NGF and its receptor TrkA are well known for a signaling to promote survival and innervation of sympathetic and sensory neurons. NGF binds to TrkA at the plasma membrane, at the axon terminal of sympathetic and sensory neurons, and activates TrkA signal transduction pathways (Marlin and Li, 2015). In addition, subcutaneous NGF injections show antidepressant effects (Overstreet et al., 2010). Thus, many studies have focused on neurotrophins as a biomarker and also as potential targets for the treatment of depression.

Decreasing structural and functional plasticity in the hippocampus and the prefrontal cortex has been thought to be associated with the gradual decline in cognitive function (Bisaz et al., 2013). The results showed that LPS-induced synaptotoxicity is associated with two dominant synaptic proteins (PSD-95 and SYP), which are involved in cognitive function and synaptic plasticity. SYP is involved in the release of presynaptic vesicles containing neurotransmitters and is used to as a marker to visualize the density of synapses (Pan et al., 2016). PSD95 is glutamatergic excitatory postsynaptic density, is involved in information storage (Gardoni et al., 2009). The increase in PSD95 expression can regulate the increase of synaptic activity. This study showed that expression of the synaptic proteins, SYP and PSD95, was decreased in the LPS group, which reflects changes in depressive behavior, while Xiaoyao Pills exerts a protective effect. These indicate that Xiaoyao Pills could restore synaptic proteins that are associated with improvement of spatial memory.

To clarify whether hippocampal neurogenesis disorder is associated with depression, we quantified BrdU-positive cell numbers in hippocampal neuron cells, and Nissl bodies staining in hippocampal CA3 area. The result showed that infection of LPS significantly decreased survival of new born cells and immature neurons which were consistent with previous reports (Monje et al., 2003).

## Conclusion

The present study demonstrated that inflammation, evoked by LPS challenge, induced depression-like behaviors, release of pro-inflammatory cytokines and the various inflammation-related mediators, a decrease of neurotrophic factor and synaptic protein, and inhibited hippocampal neurogenesis. Pre-treatment with Xiaoyao Pills significantly prevented these behavioral changes, decreased the levels of cytokines and inflammation-related mediators, increased the expression of neurotrophic factor and synaptic proteins and improved nerve damage. These findings demonstrate that Xiaoyao Pills plays an important neuroprotective role associated with inflammation induced depression, which inhibits inflammation and promotes the recovery of nerve damage.

The limitations of our study are mainly due to the characteristics of acute inflammatory stimuli that was used to evoke inflammation-induced depression. Lipopolysaccharide-induced depressive behavior summarizes the main characteristics of inflammation-induced depression, but lacks the characteristics of chronic unpredictable stress depression.

## Data Availability Statement

All data supporting the conclusions of this article are included within the article.

## Ethics Statement

All procedures were approved by the Animal Ethics Committee, Chengdu University of TCM, China (2016-11), and complied with the National Institutes of Health Guidelines for the Care and Use of Laboratory Animals.

## Author Contributions

BYS, JL, YF, XBL, ZLR, RL, and NZ conceived and designed the experiments; BYS, JL, YF, XBL, ZLR and RL performed the experiments; BYS, JL, YF, XBL, ZLR, RL, and NZ analysed the data and drafted relevant text; BYS wrote the manuscript. All authors have read and approved the final version of this manuscript.

## Funding

This work was supported by National Nature Science Foundation of China (81503277, 81473399); Education Department of Sichuan (16ZB0116); Chengdu University of Traditional Chinese Medicine (ZRQN1643).

## Conflict of Interest

The authors declare that the research was conducted in the absence of any commercial or financial relationships that could be construed as a potential conflict of interest.

## Abbreviations

XYW, Xiaoyao Pills; FLX, Fluoxetine hydrochloride; AMT, Amitriptyline; LPS, Lipopolysaccharide; IL-6, Interleukin 6; TNF-α, Tumor necrosis factor alpha; IDO, Indoleamine 2,3-dioxygenase; 5-HT, 5-hydroxytryptamine; BDNF, Brain-derived neurotrophic factor; NGF, Nerve growth factor; TrkB, Tropomyosin receptor kinase B; TrkA, Tropomyosin receptor kinase A; CREB, cAMP response element-binding protein; PSD95, postsynaptic density protein 95; SYP, synaptophysin; BrdU, 5-Bromo-2-deoxyUridine; NeuN, Vertebrate neuron-specific nuclear protein; OFT, open field test; TST, tail suspension test; FST, forced swimming test; ST, splash test; NSFT, novelty suppressed feeding test; SPT, sucrose preference test.
