# Supplementary figures and images for: Xiaoyao Pills Prevent Lipopolysaccharide-Induced Depression by Inhibiting Inflammation and Protecting Nerves
Source: Front Pharmacol. 2019 Nov 13;10:1324. doi: 10.3389/fphar.2019.01324 (PMC6863983; doi:10.3389/fphar.2019.01324)

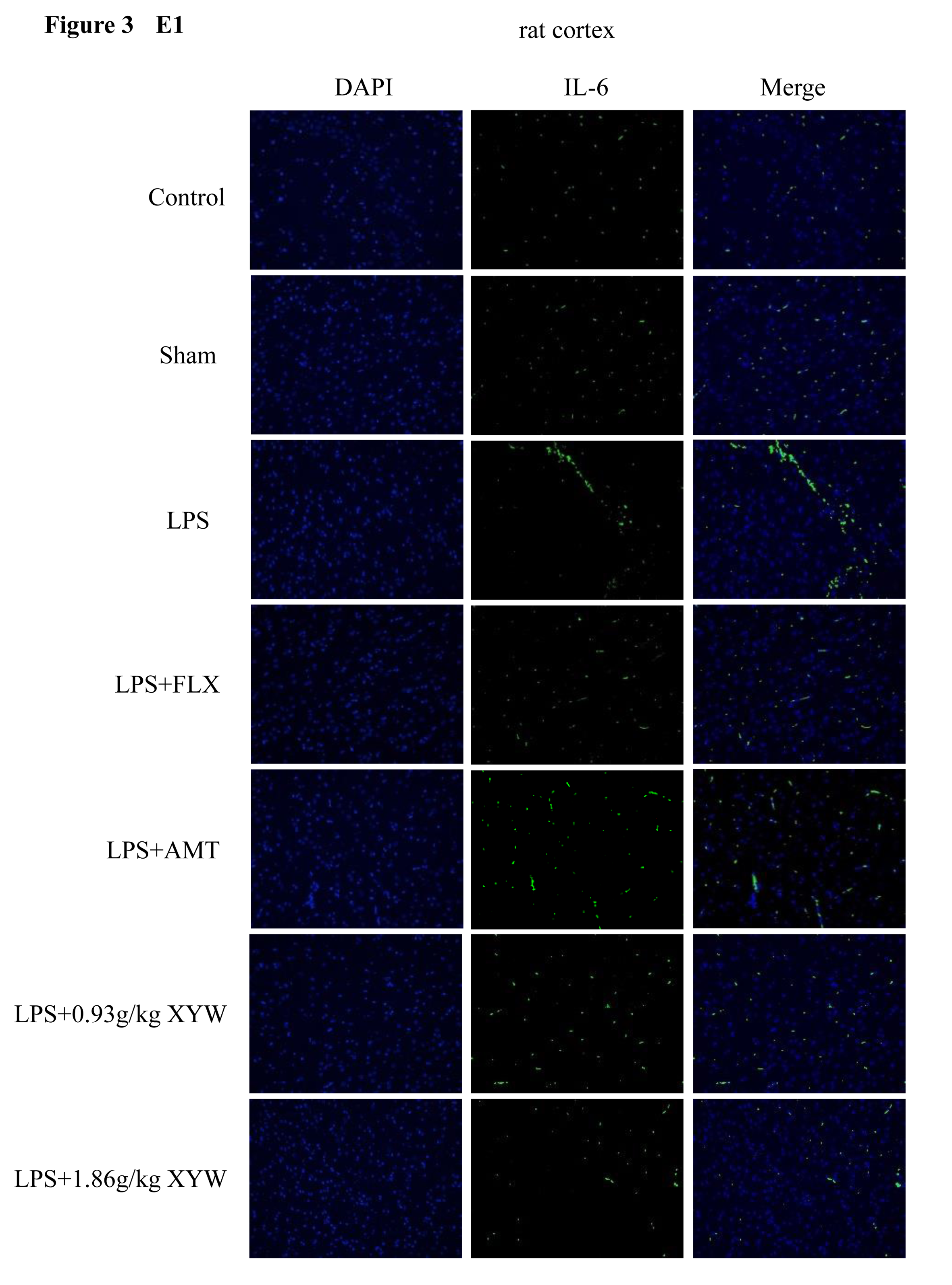

Supplement: Supplementary file 1 [file Image_1.tif]

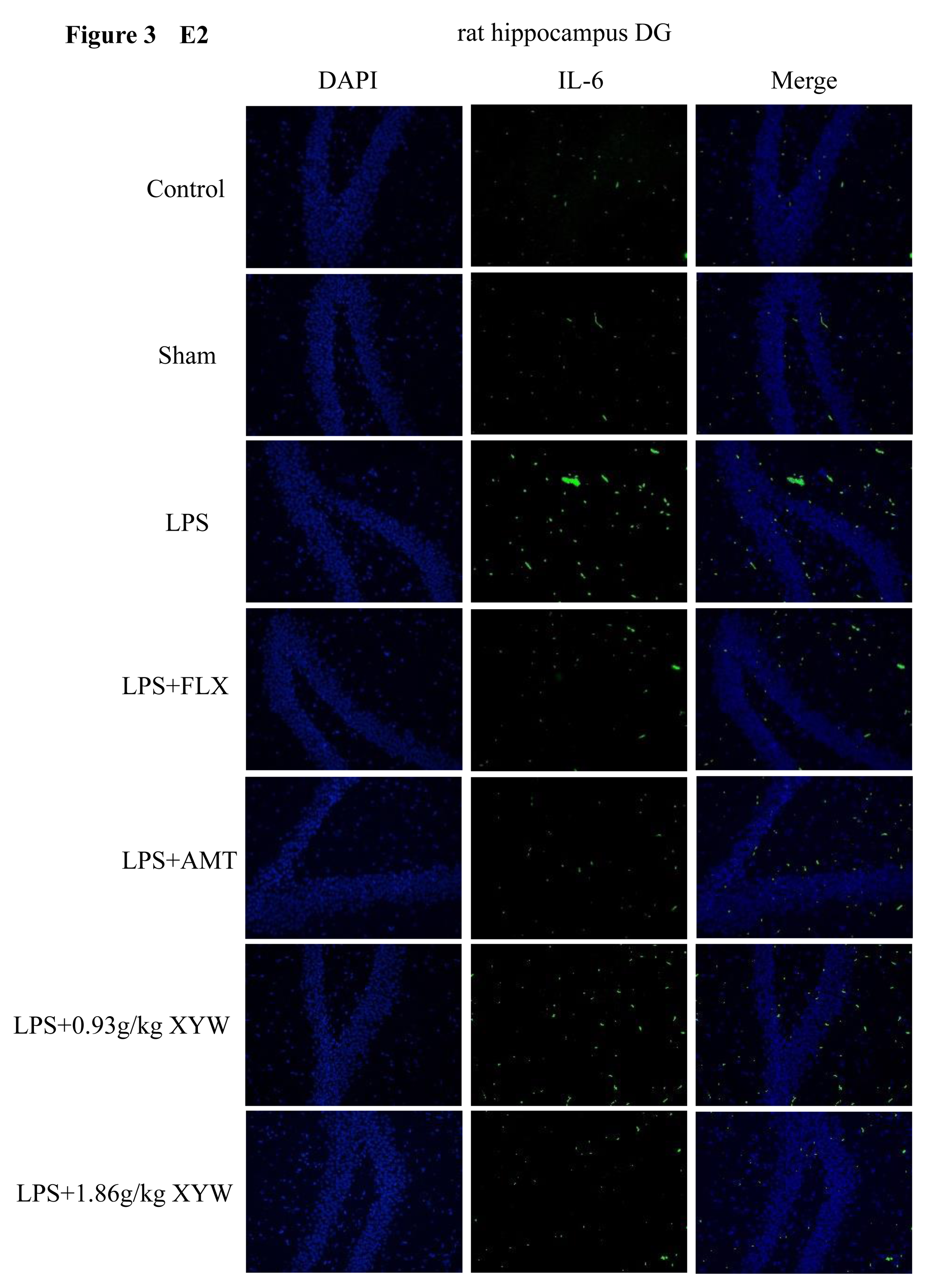

Supplement: Supplementary file 2 [file Image_2.tif]
